# Crystal structure of (*E*)-9-({[4-(di­ethyl­amino)­phen­yl]imino}­meth­yl)-2,3,6,7-tetra­hydro-1*H*,5*H*-pyrido[3,2,1-*ij*]quinolin-8-ol

**DOI:** 10.1107/S2056989016019733

**Published:** 2017-01-01

**Authors:** Md. Serajul Haque Faizi, Musheer Ahmad, Anatoly A. Kapshuk, Irina A. Golenya

**Affiliations:** a Department of Chemistry, College of Science, Sultan Qaboos University, PO Box 36, Al-Khod 123, Muscat, Sultanate of , Oman; b Department of Applied Chemistry, Aligarh Muslim University, 202002 UP, India; c Department of Chemistry, Taras Shevchenko National University of Kyiv, Vladimirska Str. 64, 01601 Kiev, Ukraine

**Keywords:** crystal structure, Schiff base, julolidine, 8-hy­droxy­julolidine-9-carboxaldehyde, *N*,*N*-diethyl-*p*-phenyl­enedi­amine, hydrogen bonding, C—H⋯π inter­actions

## Abstract

The title compound, C_23_H_29_N_3_O, was synthesized from the condensation reaction of 8-hy­droxy­julolidine-9-carbaldehyde and *N*,*N*-diethyl-*p*-phenyl­enedi­amine. The hy­droxy group forms a intra­molecular hydrogen bond to the imine N atom and generates an *S*(6) ring motif. The conformation about the C=N bond is *E*, and the aromatic ring of the julolidine moiety is inclined to the benzene ring by 3.74 (14)°. One of the fused non-aromatic rings of the julolidine moiety adopts an envelope conformation and the other has a screw-boat conformation. In the crystal, mol­ecules are linked by C—H⋯π inter­actions involving the aromatic julolidine ring, forming slabs parallel to the *bc* plane. The tricyclic fragment of the julolidine ring and the azomethine C=N bond are disordered over two sets of sites with a refined occupancy ratio of 0.773 (3):0.227 (3).

## Abstract 2

In the title compound, the hy­droxy group forms a intra­molecular hydrogen bond to the imine N atom and generates an *S*(6) ring motif. The conformation about the C=N bond is *E*, and the aromatic ring of the julolidine moiety is inclined to the benzene ring by 3.74 (14)°.

## Chemical context   

8-Hy­droxy­julolidine-9-carboxaldehyde is a well-known chromo­phore used in fluorescence chemosensors, and chemosensors with the julolidine moiety are usually soluble in aqueous solutions (Narayanaswamy & Govindaraju, 2012 ▸; Maity *et al.*, 2011 ▸; Na *et al.*, 2013 ▸; Noh *et al.*, 2013 ▸). Compounds containing a julolidine group exhibit chromogenic naked-eye detection of copper, zinc, iron and aluminium ions as well as fluoride ions (Choi *et al.*, 2015 ▸; Wang *et al.*, 2013*a* ▸,*b* ▸; Kim *et al.*, 2015 ▸; Jo *et al.*, 2015 ▸). There are many reports in the literature on 8-hy­droxy­julolidine-9-carboxaldehyde-based Schiff bases and their application as metal sensors (Park *et al.*, 2014 ▸; Lee *et al.*, 2014 ▸; Kim *et al.*, 2016 ▸). Julolidine dyes exhibit excited state intra­molecular proton transfer (Nano *et al.*, 2015 ▸), and julolidine ring-containing compounds are also used as fluorescent probes for the measurement of cell membrane viscosity.
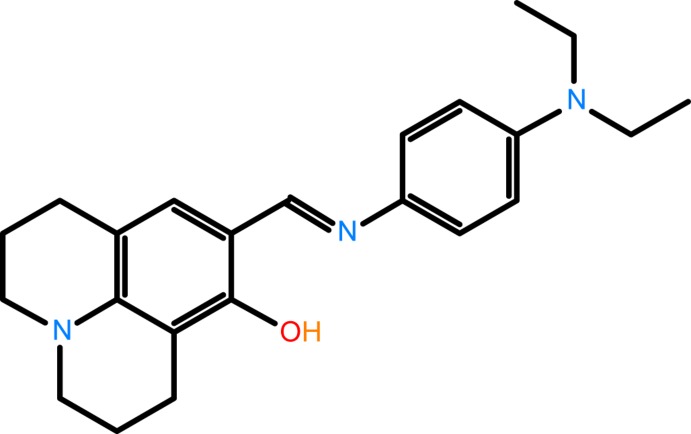


The present work is a part of an ongoing structural study of Schiff bases and their utilization in the synthesis of new organic and polynuclear coordination compounds (Faizi & Sen, 2014 ▸; Faizi *et al.*, 2015 ▸, 2016*a* ▸,*b* ▸). We report herein on the synthesis and crystal structure of a new julolidine derivative.

## Structural commentary   

**Figure 1:**
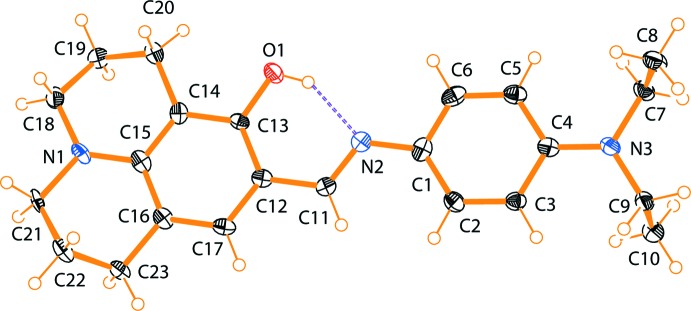
The mol­ecular structure of the title compound, showing the atom labelling. Displacement ellipsoids are drawn at the 40% probability level. The intra­molecular O—H⋯N hydrogen bond is shown as a dashed line (see Table 1 ▸). The minor component of the disordered fragment has been omitted for clarity.

The mol­ecular structure of the title compound is illustrated in Fig. 1 ▸. The conformation about the azomethine N2=C11 bond [1.285 (3) A°] is *E*, and the C14—N2—C12—C13 torsion angle is 177.86 (5)°. The mol­ecule is non-planar, with the dihedral angle between benzene ring (C1–C6) and the aromatic ring (C12–C17) of the julolidine moiety being 3.74 (14)°.

Depending on the tautomers, two types of intra­molecular hydrogen bonds are observed in Schiff bases: O—H⋯N in phenol–imine and N—H⋯O in keto–amine tautomers. The present analysis shows that the title compound exists in the phenol–imine form (Fig. 1 ▸). It exhibits an intra­molecular O—H⋯N hydrogen bond, which generates an S(6) ring motif (Fig. 1 ▸ and Table 1 ▸). This intra­molecular O—H⋯N hydrogen bond has been detected previously in julolidine derivatives (Barbero *et al.*, 2012 ▸). The C13—O1 [1.344 (2) Å] bond length is in agreement with the values reported for similar compounds, *viz*. 5-di­ethyl­amino-2-[(*E*)-(2,4-di­meth­oxy­phen­yl)imino­meth­yl]phenol and 8-{(*E*)-[(4-chloro­phen­yl)imino]­meth­yl}-1,1,7,7-tetra­methyl-1,2,3,5,6,7-hexa­hydro­pyrido[3,2,1-*ij*]quinolin-9-ol (Kantar *et al.*, 2013 ▸). One of the fused non-aromatic rings of the julolidine moiety (N1/C14/C15/C18–C20) adopts an envelope conformation while the other (N1/C15/C16/C21–C23) has a screw-boat conformation.

## Supra­molecular features   

**Figure 2:**
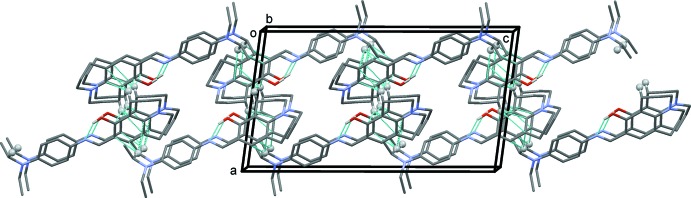
A view along the *b* axis of the crystal packing of the title compound. The C—H⋯π inter­actions are shown as dashed lines (see Table 1 ▸) and the minor component of the disordered fragment has been omitted for clarity.

In the crystal, mol­ecules are linked by C—H⋯π inter­actions (Table 1 ▸), involving the aromatic julolidine ring, forming layers lying parallel to the *bc* plane, as illustrated in Fig. 2 ▸.

## Database survey   

There are very few examples of similar compounds in the literature and, to the best of our knowledge, the new fluorescent chemosensor for the selective detection of Zn^2+^ in aqueous solution, mentioned in the *Chemical context* section (Choi *et al.*, 2015 ▸), has not been characterized crystallographically. A search of the Cambridge Structural Database (CSD, Version 5.37, update May 2016; Groom *et al.*, 2016 ▸) gave 121 hits for the julolidine moiety. Of these, six have an OH group in position 8, and four also have a C=N group in position 1. Of the latter, one compound, *viz.* 9-{[(4-chlorophen­yl)imino]­meth­yl}-1,1,7,7-tetra­methyl-2,3,6,7-tetra­hydro-1*H*,5*H*-pyrido[3,2,1-*ij*]quinolin-8-ol (CSD refcode: IGALUZ; Kantar *et al.*, 2013 ▸), resembles the title compound and also exists in the phenol–imine form with an intra­molecular O—H⋯N hydrogen bond.

## Synthesis and crystallization   

An ethano­lic solution of 8-hy­droxy­julolidine-9-carboxaldehyde (100 mg, 0.46 mmol) was added to *N*,*N*-diethyl-*p*-phenyl­enedi­amine (75 mg, 0.46 mmol) in absolute ethanol (3 ml). Two drops of HCl were added to the reaction solution and it was stirred for 30 min at room temperature. The resulting yellow precipitate was recovered by filtration, washed several times with a small portions of ice EtOH and then with diethyl ether to give 130 mg (78%) of the title compound. Colourless block-like crystals, suitable for X-ray diffraction analysis, were obtained within three days by slow evaporation of a solution in methanol.

## Refinement   

Crystal data, data collection and structure refinement details are summarized in Table 2 ▸. All the H atoms were located from difference Fourier maps but in the final cycles of refinement they were included in calculated positions and treated as riding atoms: O—H = 0.84 Å, C—H = 0.93–0.98 Å with *U*_iso_(H) = 1.5*U*_eq_(O, C-meth­yl) and 1.2*U*_eq_(C) for other H atoms. The tricyclic fragment of the julolidine ring and the azomethine C=N bond are disordered over two sets of sites with a refined occupancy ratio of 0.773 (3):0.227 (3). The non-hydrogen atoms of the major fraction were refined anisotropically while those of the minor fraction were refined isotropically, and one disordered atom, C21*A*, is probably further disordered, but this was not corrected for. The bond lengths C1—N2 and C1—N2*A* were refined with distance restraints of 1.40 (2) Å.

## Supplementary Material

Crystal structure: contains datablock(s) I, global. DOI: 10.1107/S2056989016019733/su5338sup1.cif

Supporting information file. DOI: 10.1107/S2056989016019733/su5338Isup2.cml

CCDC reference: 1521905

Additional supporting information: crystallographic information; 3D view; checkCIF report

## Figures and Tables

**Table 1:** Hydrogen-bond geometry (Å, °) *Cg*1 is the centroid of the C12–C17 ring.

*D*—H⋯*A*	*D*—H	H⋯*A*	*D*⋯*A*	*D*—H⋯*A*
O1—H1⋯N2	0.82	1.83	2.557 (4)	147
C7—H7*A*⋯*Cg*1^i^	0.97	2.79	3.574 (3)	138
C20—H20*B*⋯*Cg*1^ii^	0.97	2.62	3.521 (3)	154

Symmetry codes: (i) 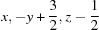; (ii) 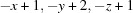.

**Table 2:** Experimental details

Crystal data	
Chemical formula	C_23_H_29_N_3_O
*M*_r_	363.49
Crystal system, space group	Monoclinic, *P*2_1_/*c*
Temperature (K)	293
*a*, *b*, *c* (Å)	11.565 (2), 8.0504 (16), 20.665 (4)
β (°)	97.68 (3)
*V* (Å^3^)	1906.7 (7)
*Z*	4
Radiation type	Mo *K*α
μ (mm^−1^)	0.08
Crystal size (mm)	0.18 × 0.14 × 0.11

Data collection	
Diffractometer	Bruker APEXII CCD
Absorption correction	Multi-scan (*SADABS*; Bruker, 2005 ▸)
*T*_min_, *T*_max_	0.894, 0.943
No. of measured, independent and observed [*I* > 2σ(*I*)] reflections	15990, 3900, 2582
*R*_int_	0.077
(sin θ/λ)_max_ (Å^−1^)	0.625

Refinement	
*R*[*F* ^2^ > 2σ(*F* ^2^)], *wR*(*F* ^2^), *S*	0.064, 0.150, 1.06
No. of reflections	3900
No. of parameters	286
No. of restraints	2
H-atom treatment	H-atom parameters constrained
Δρ_max_, Δρ_min_ (e Å^−3^)	0.33, −0.25

Computer programs: *APEX2* and *SAINT* (Bruker, 2005 ▸), *SHELXS97* (Sheldrick, 2008 ▸), *SHELXL2014* (Sheldrick, 2015 ▸), *ORTEP-3 for Windows* (Farrugia, 2012 ▸), *Mercury* (Macrae *et al.*, 2008 ▸) and *PLATON* (Spek, 2009 ▸).

## supplementary crystallographic information

### Crystal data

**Table:**

C_23_H_29_N_3_O	*F*(000) = 784
*M**_r_* = 363.49	*D*_x_ = 1.266 Mg m^−^^3^
Monoclinic, *P*2_1_/*c*	Mo *K*α radiation, λ = 0.71073 Å
*a* = 11.565 (2) Å	Cell parameters from 1274 reflections
*b* = 8.0504 (16) Å	θ = 2.8–25.3°
*c* = 20.665 (4) Å	µ = 0.08 mm^−^^1^
β = 97.68 (3)°	*T* = 293 K
*V* = 1906.7 (7) Å^3^	Block, colourless
*Z* = 4	0.18 × 0.14 × 0.11 mm

### Data collection

**Table:**

Bruker APEXII CCD diffractometer	3900 independent reflections
Radiation source: fine-focus sealed tube	2582 reflections with *I* > 2σ(*I*)
Horizontally mounted graphite crystal monochromator	*R*_int_ = 0.077
Detector resolution: 9 pixels mm^-1^	θ_max_ = 26.4°, θ_min_ = 2.7°
φ scans and ω scans with κ offset	*h* = −12→14
Absorption correction: multi-scan (SADABS; Bruker, 2005)	*k* = −10→10
*T*_min_ = 0.894, *T*_max_ = 0.943	*l* = −25→25
15990 measured reflections	

### Refinement

**Table:**

Refinement on *F*^2^	Secondary atom site location: difference Fourier map
Least-squares matrix: full	Hydrogen site location: inferred from neighbouring sites
*R*[*F*^2^ > 2σ(*F*^2^)] = 0.064	H-atom parameters constrained
*wR*(*F*^2^) = 0.150	*w* = 1/[σ^2^(*F*_o_^2^) + (0.0313*P*)^2^ + 1.5846*P*] where *P* = (*F*_o_^2^ + 2*F*_c_^2^)/3
*S* = 1.06	(Δ/σ)_max_ < 0.001
3900 reflections	Δρ_max_ = 0.33 e Å^−^^3^
286 parameters	Δρ_min_ = −0.25 e Å^−^^3^
2 restraints	Extinction correction: SHELXL2014 (Sheldrick 2015), Fc^*^=kFc[1+0.001xFc^2^λ^3^/sin(2θ)]^-1/4^
Primary atom site location: structure-invariant direct methods	Extinction coefficient: 0.0087 (10)

### Special details

**Table:**

Geometry. All esds (except the esd in the dihedral angle between two l.s. planes) are estimated using the full covariance matrix. The cell esds are taken into account individually in the estimation of esds in distances, angles and torsion angles; correlations between esds in cell parameters are only used when they are defined by crystal symmetry. An approximate (isotropic) treatment of cell esds is used for estimating esds involving l.s. planes.

### Fractional atomic coordinates and isotropic or equivalent isotropic displacement parameters (Å^2^)

**Table:**

	*x*	*y*	*z*	*U*_iso_*/*U*_eq_	Occ. (<1)
N3	0.95181 (17)	0.9756 (2)	0.12926 (9)	0.0289 (5)	
C1	0.8227 (2)	0.9508 (3)	0.31028 (11)	0.0267 (6)	
C2	0.9203 (2)	1.0455 (3)	0.30272 (11)	0.0267 (6)	
H2A	0.9582	1.1033	0.3384	0.032*	
C3	0.9622 (2)	1.0554 (3)	0.24346 (11)	0.0250 (5)	
H3A	1.0274	1.1207	0.2399	0.030*	
C4	0.9085 (2)	0.9688 (3)	0.18799 (11)	0.0242 (5)	
C5	0.8084 (2)	0.8765 (3)	0.19602 (12)	0.0287 (6)	
H5A	0.7691	0.8196	0.1606	0.034*	
C6	0.7677 (2)	0.8689 (3)	0.25534 (12)	0.0299 (6)	
H6A	0.7010	0.8069	0.2589	0.036*	
C7	0.8953 (2)	0.8891 (3)	0.07152 (11)	0.0301 (6)	
H7A	0.8613	0.7867	0.0849	0.036*	
H7B	0.9537	0.8603	0.0438	0.036*	
C8	0.8010 (2)	0.9924 (3)	0.03270 (11)	0.0334 (6)	
H8A	0.7665	0.9305	−0.0047	0.050*	
H8B	0.7422	1.0193	0.0596	0.050*	
H8C	0.8345	1.0929	0.0185	0.050*	
C9	1.0547 (2)	1.0715 (3)	0.12163 (11)	0.0289 (6)	
H9A	1.0537	1.1734	0.1466	0.035*	
H9B	1.0526	1.1015	0.0760	0.035*	
C10	1.1672 (2)	0.9792 (3)	0.14382 (12)	0.0342 (6)	
H10A	1.2325	1.0485	0.1377	0.051*	
H10B	1.1708	0.9513	0.1892	0.051*	
H10C	1.1697	0.8794	0.1186	0.051*	
O1	0.61126 (18)	0.7817 (3)	0.42135 (9)	0.0284 (6)	0.773 (3)
H1	0.6486	0.8056	0.3916	0.034*	0.773 (3)
N1	0.5791 (6)	0.9127 (8)	0.6414 (3)	0.0271 (15)	0.773 (3)
N2	0.7705 (3)	0.9226 (4)	0.36617 (15)	0.0236 (8)	0.773 (3)
C11	0.8123 (3)	0.9873 (3)	0.42118 (16)	0.0208 (7)	0.773 (3)
H11	0.8803	1.0502	0.4242	0.025*	0.773 (3)
C12	0.75428 (19)	0.9631 (3)	0.47925 (8)	0.0209 (7)	0.773 (3)
C13	0.65542 (19)	0.8636 (2)	0.47575 (8)	0.0190 (8)	0.773 (3)
C14	0.59674 (17)	0.8471 (3)	0.52987 (10)	0.0229 (7)	0.773 (3)
C15	0.6369 (2)	0.9301 (3)	0.58749 (8)	0.0236 (10)	0.773 (3)
C16	0.7358 (2)	1.0296 (3)	0.59099 (7)	0.0225 (7)	0.773 (3)
C17	0.79445 (17)	1.0461 (2)	0.53687 (9)	0.0245 (7)	0.773 (3)
H17A	0.8606	1.1127	0.5392	0.029*	0.773 (3)
C18	0.4822 (3)	0.8028 (4)	0.64386 (18)	0.0275 (8)	0.773 (3)
H18A	0.4100	0.8642	0.6333	0.033*	0.773 (3)
H18B	0.4845	0.7595	0.6878	0.033*	0.773 (3)
C19	0.4846 (3)	0.6604 (4)	0.59677 (19)	0.0279 (8)	0.773 (3)
H19A	0.4141	0.5946	0.5958	0.033*	0.773 (3)
H19B	0.5511	0.5894	0.6106	0.033*	0.773 (3)
C20	0.4932 (3)	0.7308 (4)	0.52849 (15)	0.0260 (7)	0.773 (3)
H20A	0.5014	0.6401	0.4985	0.031*	0.773 (3)
H20B	0.4221	0.7904	0.5128	0.031*	0.773 (3)
C21	0.5959 (5)	1.0430 (7)	0.6951 (2)	0.0239 (11)	0.773 (3)
H21A	0.5196	1.0810	0.7034	0.029*	0.773 (3)
H21B	0.6333	0.9908	0.7348	0.029*	0.773 (3)
C22	0.6643 (5)	1.1862 (8)	0.6814 (3)	0.0324 (13)	0.773 (3)
H22A	0.6863	1.2493	0.7212	0.039*	0.773 (3)
H22B	0.6186	1.2577	0.6500	0.039*	0.773 (3)
C23	0.7737 (3)	1.1264 (5)	0.65402 (19)	0.0279 (9)	0.773 (3)
H23A	0.8212	1.2208	0.6451	0.034*	0.773 (3)
H23B	0.8197	1.0556	0.6856	0.034*	0.773 (3)
O1A	0.8436 (7)	1.0727 (9)	0.5042 (4)	0.034 (2)*	0.227 (3)
H1AA	0.8521	1.0552	0.4660	0.051*	0.227 (3)
N1A	0.5787 (18)	0.935 (3)	0.6525 (10)	0.009 (4)*	0.227 (3)
N2A	0.8007 (11)	0.9666 (15)	0.3807 (6)	0.023 (3)*	0.227 (3)
C11A	0.7106 (9)	0.8852 (13)	0.4005 (5)	0.025 (2)*	0.227 (3)
H11A	0.6618	0.8214	0.3709	0.030*	0.227 (3)
C12A	0.6880 (9)	0.8961 (12)	0.4695 (3)	0.028 (4)*	0.227 (3)
C13A	0.7477 (7)	0.9960 (11)	0.5177 (5)	0.025 (3)*	0.227 (3)
C14A	0.7114 (9)	1.0041 (14)	0.5791 (4)	0.046 (5)*	0.227 (3)
C15A	0.6155 (9)	0.9122 (15)	0.5923 (3)	0.022 (3)*	0.227 (3)
C16A	0.5559 (7)	0.8122 (11)	0.5440 (4)	0.017 (3)*	0.227 (3)
C17A	0.5922 (8)	0.8041 (10)	0.4826 (3)	0.031 (3)*	0.227 (3)
H17B	0.5523	0.7372	0.4504	0.038*	0.227 (3)
C18A	0.4874 (14)	0.8190 (19)	0.6734 (7)	0.038 (4)*	0.227 (3)
H18C	0.5076	0.7940	0.7195	0.046*	0.227 (3)
H18D	0.4126	0.8753	0.6680	0.046*	0.227 (3)
C19A	0.4757 (11)	0.6612 (15)	0.6364 (6)	0.031 (3)*	0.227 (3)
H19C	0.4114	0.5969	0.6490	0.038*	0.227 (3)
H19D	0.5465	0.5963	0.6460	0.038*	0.227 (3)
C20A	0.4527 (11)	0.7013 (15)	0.5615 (7)	0.027 (3)*	0.227 (3)
H20C	0.3793	0.7599	0.5513	0.033*	0.227 (3)
H20D	0.4486	0.5992	0.5364	0.033*	0.227 (3)
C21A	0.607 (4)	1.042 (6)	0.694 (2)	0.131 (15)*	0.227 (3)
H21C	0.6512	0.9897	0.7323	0.157*	0.227 (3)
H21D	0.5369	1.0892	0.7081	0.157*	0.227 (3)
C22A	0.6852 (19)	1.190 (3)	0.6675 (9)	0.024 (5)*	0.227 (3)
H22C	0.6362	1.2647	0.6388	0.028*	0.227 (3)
H22D	0.7251	1.2540	0.7036	0.028*	0.227 (3)
C23A	0.7747 (14)	1.100 (2)	0.6295 (7)	0.029 (4)*	0.227 (3)
H23C	0.8229	1.1815	0.6109	0.035*	0.227 (3)
H23D	0.8251	1.0293	0.6588	0.035*	0.227 (3)

### Atomic displacement parameters (Å^2^)

**Table:**

	*U*^11^	*U*^22^	*U*^33^	*U*^12^	*U*^13^	*U*^23^
N3	0.0334 (12)	0.0315 (11)	0.0207 (10)	−0.0089 (10)	0.0000 (9)	−0.0024 (9)
C1	0.0270 (13)	0.0232 (12)	0.0307 (13)	0.0075 (11)	0.0067 (11)	0.0096 (11)
C2	0.0303 (13)	0.0252 (12)	0.0235 (12)	0.0024 (11)	0.0000 (10)	−0.0006 (10)
C3	0.0260 (13)	0.0222 (12)	0.0262 (12)	−0.0031 (10)	0.0006 (10)	0.0023 (10)
C4	0.0264 (13)	0.0191 (11)	0.0253 (12)	0.0018 (10)	−0.0027 (10)	0.0034 (10)
C5	0.0286 (13)	0.0250 (13)	0.0306 (13)	−0.0022 (11)	−0.0031 (11)	0.0027 (11)
C6	0.0250 (13)	0.0229 (12)	0.0412 (15)	−0.0017 (11)	0.0018 (11)	0.0097 (11)
C7	0.0414 (15)	0.0241 (12)	0.0241 (12)	−0.0074 (11)	0.0023 (11)	−0.0038 (11)
C8	0.0442 (16)	0.0270 (13)	0.0267 (13)	−0.0092 (12)	−0.0038 (11)	0.0001 (11)
C9	0.0368 (14)	0.0293 (13)	0.0207 (12)	−0.0080 (11)	0.0039 (10)	−0.0007 (10)
C10	0.0367 (15)	0.0369 (15)	0.0291 (13)	−0.0058 (12)	0.0041 (11)	−0.0023 (12)
O1	0.0353 (13)	0.0284 (12)	0.0212 (11)	−0.0021 (10)	0.0027 (9)	−0.0041 (9)
N1	0.038 (2)	0.021 (3)	0.021 (3)	0.0025 (16)	0.0033 (19)	−0.0086 (18)
N2	0.0244 (17)	0.0194 (16)	0.0268 (17)	0.0040 (14)	0.0029 (14)	0.0021 (13)
C11	0.0210 (15)	0.0143 (14)	0.0269 (18)	0.0029 (12)	0.0022 (13)	0.0026 (13)
C12	0.0241 (17)	0.0124 (14)	0.0252 (17)	0.0024 (13)	−0.0008 (13)	0.0028 (13)
C13	0.0208 (19)	0.0129 (15)	0.0221 (16)	0.0025 (16)	−0.0012 (13)	−0.0030 (12)
C14	0.0276 (18)	0.0196 (16)	0.0222 (17)	0.0055 (14)	0.0054 (14)	0.0048 (13)
C15	0.033 (2)	0.0161 (17)	0.0201 (17)	0.0108 (16)	−0.0008 (14)	0.0040 (13)
C16	0.0298 (18)	0.0170 (16)	0.0192 (16)	0.0081 (15)	−0.0022 (15)	0.0061 (14)
C17	0.0245 (17)	0.0188 (15)	0.0278 (17)	−0.0010 (14)	−0.0060 (15)	0.0006 (14)
C18	0.0267 (18)	0.034 (2)	0.0224 (19)	0.0030 (15)	0.0054 (15)	0.0011 (17)
C19	0.0286 (18)	0.0233 (17)	0.032 (2)	0.0009 (14)	0.0030 (16)	0.0018 (16)
C20	0.0325 (18)	0.0235 (16)	0.0219 (16)	0.0062 (15)	0.0036 (14)	0.0007 (14)
C21	0.035 (2)	0.0291 (19)	0.0078 (14)	0.0111 (15)	0.0027 (12)	−0.0008 (13)
C22	0.047 (3)	0.031 (2)	0.018 (2)	0.003 (2)	0.000 (2)	−0.005 (2)
C23	0.035 (2)	0.0259 (19)	0.021 (2)	−0.0021 (15)	−0.0018 (17)	−0.0045 (17)

### Geometric parameters (Å, º)

**Table:**

N3—C4	1.375 (3)	C19—C20	1.536 (4)
N3—C9	1.445 (3)	C19—H19A	0.9700
N3—C7	1.458 (3)	C19—H19B	0.9700
C1—C2	1.388 (3)	C20—H20A	0.9700
C1—C6	1.391 (3)	C20—H20B	0.9700
C1—N2	1.392 (4)	C21—C22	1.447 (9)
C1—N2A	1.516 (12)	C21—H21A	0.9700
C2—C3	1.378 (3)	C21—H21B	0.9700
C2—H2A	0.9300	C22—C23	1.532 (7)
C3—C4	1.412 (3)	C22—H22A	0.9700
C3—H3A	0.9300	C22—H22B	0.9700
C4—C5	1.404 (3)	C23—H23A	0.9700
C5—C6	1.372 (3)	C23—H23B	0.9700
C5—H5A	0.9300	O1A—C13A	1.331 (11)
C6—H6A	0.9300	O1A—H1AA	0.8200
C7—C8	1.514 (3)	N1A—C21A	1.23 (4)
C7—H7A	0.9700	N1A—C15A	1.38 (2)
C7—H7B	0.9700	N1A—C18A	1.51 (3)
C8—H8A	0.9600	N2A—C11A	1.341 (16)
C8—H8B	0.9600	C11A—C12A	1.485 (12)
C8—H8C	0.9600	C11A—H11A	0.9300
C9—C10	1.515 (3)	C12A—C13A	1.3900
C9—H9A	0.9700	C12A—C17A	1.3900
C9—H9B	0.9700	C13A—C14A	1.3900
C10—H10A	0.9600	C14A—C15A	1.3900
C10—H10B	0.9600	C14A—C23A	1.421 (16)
C10—H10C	0.9600	C15A—C16A	1.3900
O1—C13	1.344 (2)	C16A—C17A	1.3900
O1—H1	0.8200	C16A—C20A	1.571 (14)
N1—C15	1.381 (6)	C17A—H17B	0.9300
N1—C18	1.434 (8)	C18A—C19A	1.479 (18)
N1—C21	1.521 (7)	C18A—H18C	0.9700
N2—C11	1.285 (5)	C18A—H18D	0.9700
C11—C12	1.464 (3)	C19A—C20A	1.568 (17)
C11—H11	0.9300	C19A—H19C	0.9700
C12—C13	1.3900	C19A—H19D	0.9700
C12—C17	1.3900	C20A—H20C	0.9700
C13—C14	1.3900	C20A—H20D	0.9700
C14—C15	1.3900	C21A—C22A	1.64 (5)
C14—C20	1.517 (4)	C21A—H21C	0.9700
C15—C16	1.3900	C21A—H21D	0.9700
C16—C17	1.3900	C22A—C23A	1.56 (2)
C16—C23	1.531 (4)	C22A—H22C	0.9700
C17—H17A	0.9300	C22A—H22D	0.9700
C18—C19	1.506 (5)	C23A—H23C	0.9700
C18—H18A	0.9700	C23A—H23D	0.9700
C18—H18B	0.9700		
			
C4—N3—C9	121.33 (19)	C14—C20—H20A	109.4
C4—N3—C7	121.8 (2)	C19—C20—H20A	109.4
C9—N3—C7	116.83 (19)	C14—C20—H20B	109.4
C2—C1—C6	117.3 (2)	C19—C20—H20B	109.4
C2—C1—N2	129.3 (3)	H20A—C20—H20B	108.0
C6—C1—N2	113.4 (2)	C22—C21—N1	115.2 (5)
C2—C1—N2A	107.8 (5)	C22—C21—H21A	108.5
C6—C1—N2A	134.9 (5)	N1—C21—H21A	108.5
C3—C2—C1	121.3 (2)	C22—C21—H21B	108.5
C3—C2—H2A	119.3	N1—C21—H21B	108.5
C1—C2—H2A	119.3	H21A—C21—H21B	107.5
C2—C3—C4	121.6 (2)	C21—C22—C23	108.8 (5)
C2—C3—H3A	119.2	C21—C22—H22A	109.9
C4—C3—H3A	119.2	C23—C22—H22A	109.9
N3—C4—C5	121.9 (2)	C21—C22—H22B	109.9
N3—C4—C3	121.6 (2)	C23—C22—H22B	109.9
C5—C4—C3	116.5 (2)	H22A—C22—H22B	108.3
C6—C5—C4	121.1 (2)	C16—C23—C22	108.5 (3)
C6—C5—H5A	119.5	C16—C23—H23A	110.0
C4—C5—H5A	119.5	C22—C23—H23A	110.0
C5—C6—C1	122.2 (2)	C16—C23—H23B	110.0
C5—C6—H6A	118.9	C22—C23—H23B	110.0
C1—C6—H6A	118.9	H23A—C23—H23B	108.4
N3—C7—C8	112.5 (2)	C13A—O1A—H1AA	109.5
N3—C7—H7A	109.1	C21A—N1A—C15A	130 (3)
C8—C7—H7A	109.1	C21A—N1A—C18A	111 (3)
N3—C7—H7B	109.1	C15A—N1A—C18A	119.1 (16)
C8—C7—H7B	109.1	C11A—N2A—C1	119.3 (10)
H7A—C7—H7B	107.8	N2A—C11A—C12A	120.5 (10)
C7—C8—H8A	109.5	N2A—C11A—H11A	119.7
C7—C8—H8B	109.5	C12A—C11A—H11A	119.7
H8A—C8—H8B	109.5	C13A—C12A—C17A	120.0
C7—C8—H8C	109.5	C13A—C12A—C11A	126.0 (8)
H8A—C8—H8C	109.5	C17A—C12A—C11A	113.8 (8)
H8B—C8—H8C	109.5	O1A—C13A—C12A	117.8 (8)
N3—C9—C10	113.1 (2)	O1A—C13A—C14A	122.1 (8)
N3—C9—H9A	109.0	C12A—C13A—C14A	120.0
C10—C9—H9A	109.0	C15A—C14A—C13A	120.0
N3—C9—H9B	109.0	C15A—C14A—C23A	119.5 (9)
C10—C9—H9B	109.0	C13A—C14A—C23A	120.4 (9)
H9A—C9—H9B	107.8	N1A—C15A—C14A	117.2 (11)
C9—C10—H10A	109.5	N1A—C15A—C16A	122.6 (11)
C9—C10—H10B	109.5	C14A—C15A—C16A	120.0
H10A—C10—H10B	109.5	C17A—C16A—C15A	120.0
C9—C10—H10C	109.5	C17A—C16A—C20A	121.0 (7)
H10A—C10—H10C	109.5	C15A—C16A—C20A	118.9 (7)
H10B—C10—H10C	109.5	C16A—C17A—C12A	120.0
C13—O1—H1	109.5	C16A—C17A—H17B	120.0
C15—N1—C18	123.8 (4)	C12A—C17A—H17B	120.0
C15—N1—C21	119.4 (5)	C19A—C18A—N1A	113.6 (13)
C18—N1—C21	115.1 (5)	C19A—C18A—H18C	108.8
C11—N2—C1	120.9 (3)	N1A—C18A—H18C	108.8
N2—C11—C12	120.8 (3)	C19A—C18A—H18D	108.8
N2—C11—H11	119.6	N1A—C18A—H18D	108.8
C12—C11—H11	119.6	H18C—C18A—H18D	107.7
C13—C12—C17	120.0	C18A—C19A—C20A	109.0 (11)
C13—C12—C11	119.86 (18)	C18A—C19A—H19C	109.9
C17—C12—C11	120.06 (18)	C20A—C19A—H19C	109.9
O1—C13—C14	117.02 (18)	C18A—C19A—H19D	109.9
O1—C13—C12	122.97 (18)	C20A—C19A—H19D	109.9
C14—C13—C12	120.0	H19C—C19A—H19D	108.3
C13—C14—C15	120.0	C19A—C20A—C16A	108.1 (9)
C13—C14—C20	120.86 (18)	C19A—C20A—H20C	110.1
C15—C14—C20	119.03 (18)	C16A—C20A—H20C	110.1
N1—C15—C14	120.0 (3)	C19A—C20A—H20D	110.1
N1—C15—C16	120.0 (3)	C16A—C20A—H20D	110.1
C14—C15—C16	120.0	H20C—C20A—H20D	108.4
C17—C16—C15	120.0	N1A—C21A—C22A	112 (3)
C17—C16—C23	121.4 (2)	N1A—C21A—H21C	109.2
C15—C16—C23	118.6 (2)	C22A—C21A—H21C	109.2
C16—C17—C12	120.0	N1A—C21A—H21D	109.2
C16—C17—H17A	120.0	C22A—C21A—H21D	109.2
C12—C17—H17A	120.0	H21C—C21A—H21D	107.9
N1—C18—C19	111.4 (3)	C23A—C22A—C21A	105 (2)
N1—C18—H18A	109.3	C23A—C22A—H22C	110.6
C19—C18—H18A	109.3	C21A—C22A—H22C	110.6
N1—C18—H18B	109.3	C23A—C22A—H22D	110.6
C19—C18—H18B	109.3	C21A—C22A—H22D	110.6
H18A—C18—H18B	108.0	H22C—C22A—H22D	108.8
C18—C19—C20	108.8 (3)	C14A—C23A—C22A	108.1 (14)
C18—C19—H19A	109.9	C14A—C23A—H23C	110.1
C20—C19—H19A	109.9	C22A—C23A—H23C	110.1
C18—C19—H19B	109.9	C14A—C23A—H23D	110.1
C20—C19—H19B	109.9	C22A—C23A—H23D	110.1
H19A—C19—H19B	108.3	H23C—C23A—H23D	108.4
C14—C20—C19	111.0 (2)		
			
C6—C1—C2—C3	−1.2 (3)	N1—C18—C19—C20	−53.5 (4)
N2—C1—C2—C3	178.0 (3)	C13—C14—C20—C19	149.3 (2)
N2A—C1—C2—C3	179.9 (5)	C15—C14—C20—C19	−26.9 (3)
C1—C2—C3—C4	−0.6 (4)	C18—C19—C20—C14	54.4 (3)
C9—N3—C4—C5	179.8 (2)	C15—N1—C21—C22	5.9 (8)
C7—N3—C4—C5	0.9 (3)	C18—N1—C21—C22	−159.6 (5)
C9—N3—C4—C3	0.2 (3)	N1—C21—C22—C23	−46.1 (6)
C7—N3—C4—C3	−178.7 (2)	C17—C16—C23—C22	139.8 (3)
C2—C3—C4—N3	−178.5 (2)	C15—C16—C23—C22	−36.9 (4)
C2—C3—C4—C5	1.9 (3)	C21—C22—C23—C16	60.4 (5)
N3—C4—C5—C6	178.9 (2)	C2—C1—N2A—C11A	−179.7 (9)
C3—C4—C5—C6	−1.5 (3)	C6—C1—N2A—C11A	1.6 (15)
C4—C5—C6—C1	−0.2 (4)	C1—N2A—C11A—C12A	179.0 (8)
C2—C1—C6—C5	1.5 (3)	N2A—C11A—C12A—C13A	5.3 (14)
N2—C1—C6—C5	−177.8 (2)	N2A—C11A—C12A—C17A	−179.6 (9)
N2A—C1—C6—C5	−179.9 (7)	C17A—C12A—C13A—O1A	175.5 (8)
C4—N3—C7—C8	87.5 (3)	C11A—C12A—C13A—O1A	−9.7 (10)
C9—N3—C7—C8	−91.5 (3)	C17A—C12A—C13A—C14A	0.0
C4—N3—C9—C10	83.4 (3)	C11A—C12A—C13A—C14A	174.9 (10)
C7—N3—C9—C10	−97.6 (2)	O1A—C13A—C14A—C15A	−175.3 (9)
C2—C1—N2—C11	−0.4 (4)	C12A—C13A—C14A—C15A	0.0
C6—C1—N2—C11	178.8 (2)	O1A—C13A—C14A—C23A	2.1 (12)
C1—N2—C11—C12	177.4 (2)	C12A—C13A—C14A—C23A	177.3 (12)
N2—C11—C12—C13	2.7 (3)	C21A—N1A—C15A—C14A	11 (4)
N2—C11—C12—C17	−174.1 (2)	C18A—N1A—C15A—C14A	−170.4 (12)
C17—C12—C13—O1	179.2 (2)	C21A—N1A—C15A—C16A	−164 (3)
C11—C12—C13—O1	2.4 (2)	C18A—N1A—C15A—C16A	14 (2)
C17—C12—C13—C14	0.0	C13A—C14A—C15A—N1A	−175.3 (14)
C11—C12—C13—C14	−176.8 (2)	C23A—C14A—C15A—N1A	7.3 (13)
O1—C13—C14—C15	−179.2 (2)	C13A—C14A—C15A—C16A	0.0
C12—C13—C14—C15	0.0	C23A—C14A—C15A—C16A	−177.4 (12)
O1—C13—C14—C20	4.6 (2)	N1A—C15A—C16A—C17A	175.1 (15)
C12—C13—C14—C20	−176.1 (2)	C14A—C15A—C16A—C17A	0.0
C18—N1—C15—C14	4.7 (7)	N1A—C15A—C16A—C20A	−8.7 (15)
C21—N1—C15—C14	−159.5 (4)	C14A—C15A—C16A—C20A	176.3 (9)
C18—N1—C15—C16	−174.9 (4)	C15A—C16A—C17A—C12A	0.0
C21—N1—C15—C16	20.9 (7)	C20A—C16A—C17A—C12A	−176.2 (9)
C13—C14—C15—N1	−179.6 (4)	C13A—C12A—C17A—C16A	0.0
C20—C14—C15—N1	−3.3 (4)	C11A—C12A—C17A—C16A	−175.4 (9)
C13—C14—C15—C16	0.0	C21A—N1A—C18A—C19A	−162 (3)
C20—C14—C15—C16	176.2 (2)	C15A—N1A—C18A—C19A	19 (2)
N1—C15—C16—C17	179.6 (4)	N1A—C18A—C19A—C20A	−54.2 (16)
C14—C15—C16—C17	0.0	C18A—C19A—C20A—C16A	57.0 (13)
N1—C15—C16—C23	−3.7 (4)	C17A—C16A—C20A—C19A	149.1 (8)
C14—C15—C16—C23	176.8 (3)	C15A—C16A—C20A—C19A	−27.1 (11)
C15—C16—C17—C12	0.0	C15A—N1A—C21A—C22A	10 (5)
C23—C16—C17—C12	−176.7 (3)	C18A—N1A—C21A—C22A	−169 (2)
C13—C12—C17—C16	0.0	N1A—C21A—C22A—C23A	−44 (4)
C11—C12—C17—C16	176.8 (2)	C15A—C14A—C23A—C22A	−42.8 (15)
C15—N1—C18—C19	25.3 (7)	C13A—C14A—C23A—C22A	139.8 (12)
C21—N1—C18—C19	−169.9 (4)	C21A—C22A—C23A—C14A	58 (2)

### Hydrogen-bond geometry (Å, º)

Cg1 is the centroid of the C12–C17 ring.

**Table:**

*D*—H···*A*	*D*—H	H···*A*	*D*···*A*	*D*—H···*A*
O1—H1···N2	0.82	1.83	2.557 (4)	147
C7—H7*A*···*Cg*1^i^	0.97	2.79	3.574 (3)	138
C20—H20*B*···*Cg*1^ii^	0.97	2.62	3.521 (3)	154

Symmetry codes: (i) *x*, −*y*+3/2, *z*−1/2; (ii) −*x*+1, −*y*+2, −*z*+1.
